# Explainable Boosting Machine in Sepsis Prediction Using Platelet Metabolomics: An Interpretable Machine Learning Approach

**DOI:** 10.3390/diagnostics16111643

**Published:** 2026-05-27

**Authors:** Emek Guldogan, Burak Yagin, Yavuz Korkmaz, Sarah A. Alzakari, Amal K. Alkhalifa, Fahaid Al-Hashem, Fatma Hilal Yagin

**Affiliations:** 1 Department of Biostatistics, and Medical Informatics, Faculty of Medicine, Inonu University, 44280 Malatya, Türkiye; 2 Department of Family Medicine, Faculty of Medicine, Malatya Turgut Ozal University, 44210 Malatya, Türkiye; 3 Department of Computer Sciences, College of Computer and Information Sciences, Princess Nourah bint Abdulrahman University, P.O. Box 84428, Riyadh 11671, Saudi Arabia; 4 Department of Physiology, College of Medicine, King Khalid University, Abha 61421, Saudi Arabia; 5 Department of Biostatistics, Faculty of Medicine, Malatya Turgut Ozal University, 44210 Malatya, Türkiye

**Keywords:** sepsis, metabolomics, machine learning, explainable boosting machine, platelet metabolism, biomarkers, clinical decision support

## Abstract

**Background:** Sepsis remains a leading cause of mortality in emergency and intensive care settings, with early diagnosis representing a critical determinant of patient outcomes. Despite advances in biomarker discovery, integrating platelet-derived metabolic signatures with explainable machine learning frameworks for sepsis prediction remains underexplored. The clinical adoption of predictive models has been hindered by the “black box” nature of conventional algorithms, limiting clinician trust and understanding. **Objective:** This study aimed to evaluate and validate an interpretable machine learning model utilizing platelet metabolomics data for accurate sepsis prediction while providing clinically meaningful explanations of the underlying metabolic disturbances that could inform therapeutic decision-making. **Methods:** We analyzed metabolomics data, comprising 25 sepsis patients diagnosed according to Sepsis-3 criteria and 14 age- and gender-matched non-sepsis from the emergency department. Platelet metabolite profiles were obtained via quantitative ^1^H-NMR spectroscopy. Five machine learning algorithms were evaluated: Explainable Boosting Machine (EBM), Support Vector Machine (SVM), Logistic Regression (LR), Gradient Boosting Machine (GBM), and AdaBoost. Three biologically motivated metabolite ratios (adenosine triphosphate/adenosine diphosphate (ATP/ADP), ATP/adenosine monophosphate (AMP), Glutamine/Glutamate) were derived as additional features, yielding 22 candidate variables. Models were evaluated using a fully nested leave-one-out cross-validation (LOOCV) framework in which log transformation, KNN imputation, BorderlineSMOTE class balancing, and hyperparameter optimisation were performed exclusively within each training fold. Global and local interpretability analyses were performed to identify discriminative metabolites. **Results:** EBM achieved the highest ROC-AUC (0.864; 95% CI: 0.736–1.000), the highest PR-AUC (0.902; 95% CI: 0.783–0.997), and the best Brier score (0.189; 95% CI: 0.130–0.258) among all evaluated models, with sensitivity 0.880 (95% CI: 0.640–1.000; TP = 22/25) and specificity 0.714 (95% CI: 0.357–1.000; TN = 10/14). Global feature importance identified Carnitine, myo-Inositol, ADP, and O-Phosphoethanolamine as the leading single-feature predictors, alongside three pairwise interaction terms reflecting non-additive energy–amino acid metabolic relationships. Local explanations demonstrated that the ADP–Creatine interaction, Glutamine, and myo-Inositol drove correct sepsis classification in a representative true positive case. **Conclusions:** The EBM model demonstrated the highest discriminative performance and best calibration among all evaluated models, providing transparent mechanistic insights through global feature importance, and patient-level local explanations. These findings position the proposed framework as a proof-of-concept warranting external validation in larger, multi-centre cohorts before any clinical application is considered.

## 1. Introduction

Sepsis, defined as life-threatening organ dysfunction caused by a dysregulated host response to infection, represents one of the most formidable challenges in contemporary critical care medicine [1]. Despite substantial advances in our understanding of sepsis pathophysiology and therapeutic interventions over the past decades, the condition continues to impose a devastating burden on global healthcare systems, accounting for approximately 11 million deaths annually—representing nearly 20% of all global deaths [2]. The economic impact is equally staggering, with sepsis-related hospitalizations costing healthcare systems billions of dollars annually, while survivors frequently experience long-term physical, cognitive, and psychological sequelae that profoundly affect quality of life. Post-sepsis syndrome affects a significant portion of survivors, and persistent cognitive impairment, physical disability, and increased susceptibility to recurrent infections are major determinants of long-term morbidity. The economic burden extends beyond acute hospitalization, with estimated annual costs exceeding billions of dollars in the United States alone, underscoring the urgent need for early diagnostic and prognostic tools [3].

The complexity of sepsis lies not only in its heterogeneous clinical presentation but also in the profound and dynamic metabolic derangements that accompany the systemic inflammatory response, rendering early diagnosis and risk stratification particularly challenging endeavors. Traditional biomarkers such as procalcitonin, C-reactive protein, and lactate, while clinically useful, exhibit limited sensitivity and specificity for early sepsis detection, particularly in distinguishing sepsis from non-infectious systemic inflammatory response syndrome (SIRS) [4]. The “golden hour” concept in sepsis management underscores the critical importance of early recognition; each hour of delay in appropriate antibiotic therapy is associated with measurably increased mortality, creating an urgent need for more accurate and rapid diagnostic tools.

The advent of metabolomics has revolutionized our capacity to interrogate the biochemical landscape of critical illness at an unprecedented level of granularity. By systematically profiling small-molecule metabolites in biological specimens, metabolomics offers a unique window into the dynamic alterations in cellular metabolism that characterize disease states [5]. In the context of sepsis, metabolomic investigations have revealed perturbations across multiple interconnected pathways, including energy metabolism, amino acid homeostasis, lipid utilization, and oxidative stress responses, collectively reflecting the profound metabolic crisis imposed by overwhelming infection [6]. These metabolic signatures hold considerable promise as diagnostic and prognostic biomarkers, potentially enabling earlier detection and more precise risk stratification than conventional clinical parameters alone.

Platelets, traditionally viewed as cellular mediators of hemostasis, have increasingly been recognized as active and sophisticated participants in the innate immune response to infection. Beyond their canonical roles in coagulation, platelets exhibit remarkable metabolic machinery that responds dynamically to inflammatory stimuli and pathogen recognition [4]. During sepsis, platelet activation, consumption, and dysfunction occur in concert with dramatic alterations in platelet metabolic profiles, reflecting both intrinsic metabolic reprogramming and the broader systemic derangements characteristic of the septic state. Consequently, platelet-derived metabolites represent an attractive and relatively underexplored source of biomarkers for sepsis diagnosis and monitoring, potentially offering insights into both the inflammatory and coagulation cascades simultaneously activated in sepsis.

The application of machine learning algorithms to clinical prediction problems has yielded substantial improvements in diagnostic accuracy across numerous medical domains. Gradient boosting methods have demonstrated particular efficacy in handling the complex, high-dimensional, and often imbalanced data typical of metabolomic studies [7]. However, the clinical adoption of machine learning-based prediction tools has been significantly hindered by concerns regarding interpretability; the so-called “black box” nature of many sophisticated algorithms limits clinicians’ ability to understand, trust, and appropriately apply model predictions in clinical decision-making [8]. This limitation is especially salient in critical care settings, where treatment decisions must be made rapidly under uncertainty and where clinicians require not merely accurate predictions but also mechanistic insights that can inform therapeutic strategies and facilitate shared decision-making with patients and families.

The Explainable Boosting Machine (EBM), developed by Microsoft Research, represents a compelling solution to this interpretability challenge. As a glass-box model built upon the foundation of generalized additive models (GAMs), EBM combines the predictive power of modern machine learning with the interpretability traditionally associated with linear models. By decomposing complex predictions into interpretable univariate shape functions and pairwise interaction terms, EBM enables clinicians to visualize and comprehend the contribution of individual features to model outputs while maintaining predictive performance comparable to state-of-the-art black-box ensemble methods [9]. This inherent interpretability positions EBM as a particularly attractive framework for clinical applications, where transparency, mechanistic plausibility, and regulatory compliance are paramount considerations. The regulatory landscape for artificial intelligence in healthcare increasingly emphasizes explainability as a prerequisite for clinical deployment, with the United States Food and Drug Administration and European Medicines Agency both highlighting the importance of transparent decision-making processes in medical devices. Inherently interpretable models such as EBM may face fewer regulatory hurdles compared to black-box alternatives requiring extensive post hoc explanation validation [10,11,12].

In a previous study, XGBoost, LightGBM, and KTBoost were applied to a metabolomic dataset using SHAP-based post-annotation methods and 5-fold cross-validation without a nested design; this approach involved prior validation with SMOTE-Tomek oversampling and hyperparameter selection, resulting in selection optimism that inflated performance estimates in small samples [13]. Furthermore, post-annotation methods like SHAP provide local linear approximations rather than exact representations of model behavior, and calibration metrics are not reported. This study addresses these limitations by implementing a fully nested LOOCV framework with an inherently interpretable transparent box model (EBM), biologically motivated feature engineering, and comprehensive reporting of calibration and precision-recall performance. Despite the growing body of literature on metabolomics-based sepsis biomarkers and machine learning applications in critical care, a significant knowledge gap persists regarding the integration of platelet-specific metabolomic signatures with inherently interpretable machine learning frameworks. Previous studies have predominantly employed black-box algorithms with post hoc explanation techniques, which may not fully capture the complex, non-linear relationships between metabolites and sepsis status while providing the transparency required for clinical translation.

The present study addresses this methodological gap through a secondary machine learning analysis of publicly available platelet metabolomics data. Specifically, we pursued four objectives: (1) to evaluate five machine learning algorithms spanning inherently interpretable and black-box architectures for discriminating sepsis patients from emergency department controls, using a fully nested LOOCV framework in which all preprocessing, class balancing, and hyperparameter optimisation are confined to the training partition of each fold; (2) to identify the most discriminative platelet metabolites through global EBM feature importance analysis, leveraging the model’s exact shape functions rather than post hoc approximations; (3) to generate patient-level mechanistic explanations using EBM’s additive local decomposition for representative; and (4) to interpret identified metabolite signatures in the context of established sepsis pathophysiology, generating hypotheses for targeted investigation.

## 2. Materials and Methods

### 2.1. Study Design and Participant

This study analyzed publicly available (secondary analysis) metabolomics data from the National Metabolomics Data Repository (project ID: ST001294, www.metabolomicsworkbench.org; 2 December 2025) following approval by the Inonu University Health Sciences Non-Interventional Clinical Research Ethics Committee (2026/9231). We enrolled 39 participants comprising 25 sepsis patients and 14 non-sepsis (emergency department patients without chronic conditions or medications known to affect platelet function) from the emergency department (ED). Non-sepsis patients were age- and gender-comparable to sepsis patients, who presented with a median SOFA score of 4.5 (IQR: 3.0–8.5). Sepsis patients met the following criteria: confirmed or suspected infection with acute SOFA score increase ≥2 points, presence of ≥2 SIRS criteria, age ≥18 years, lactate ≥2.0 mmol/L, and enrollment within two hours of initiating quantitative resuscitation. Non-sepsis participants were ED patients without chronic conditions or medications affecting platelet function. Exclusion criteria included non-sepsis primary diagnoses, do-not-resuscitate status, inter-hospital transfers with prior sepsis treatment, and pre-enrollment cardiopulmonary resuscitation. This diagnostic accuracy study was conducted and reported in accordance with the Transparent Reporting of a Multivariable Prediction Model for Individual Prognosis or Diagnosis (TRIPOD) statement [14].

### 2.2. Sample Processing and NMR Analysis

Whole blood (12 mL) was collected in K2-EDTA tubes and processed via two-stage centrifugation: 200× *g* for 6 min at room temperature (platelet-rich plasma separation), followed by 4500× *g* for 5 min at 4 °C (platelet pelleting). Platelet pellets were resuspended in residual plasma (~0.25 mL) and subjected to methanol-chloroform extraction after two freeze–thaw cycles. Quantitative ^1^H-NMR spectroscopy was employed for metabolite profiling, leveraging its non-destructive nature, excellent reproducibility, and superior detection of polar metabolites including carbohydrates, organic acids, and polyols—compounds often challenging to analyze via mass spectrometry platforms. Quantitative ^1^H-NMR spectroscopy yielded 19 platelet metabolites: adenosine diphosphate (ADP), adenosine monophosphate (AMP), adenosine triphosphate (ATP), alanine, carnitine, choline, creatine, formate, guanosine triphosphate (GTP), glucose, glutamate, glutamine, glycine, lactate, O-acetylcholine, O-phosphocholine, O-phosphoethanolamine, taurine, and myo-inositol.

### 2.3. Data Preprocessing

Raw metabolite concentrations were log-transformed [log_1_(x + 1)] prior to any further processing to stabilize variance across the wide concentration ranges typical of quantitative ^1^H-NMR data. Missing values were then handled with K-Nearest Neighbors imputation (k = 5, Euclidean distance metric) [15]. Feature-wise standardization (zero mean, unit variance) was applied after imputation. All three of these transformations were fit on the training fold in each cross-validation iteration and applied to the held-out test sample using the training-fold parameters, with no refitting on test data. To enrich the metabolite feature space, three biologically motivated ratios were computed and appended to the 19 NMR-quantified features, yielding 22 candidate features: (1) ATP/ADP ratio, reflecting cellular energy charge and mitochondrial oxidative phosphorylation capacity; (2) ATP/AMP ratio, an indicator of AMPK activation status; and (3) Glutamine/Glutamate ratio, a marker of redox balance and immune cell metabolic demand. To address the class imbalance (25 sepsis patients vs. 14 controls), BorderlineSMOTE [16] was applied within the training fold. BorderlineSMOTE restricts synthetic sample generation to minority-class instances near the decision boundary, a more targeted strategy than standard SMOTE for datasets where borderline cases drive misclassification. In each of the 39 LOOCV training folds (~38 samples: ~24 sepsis, ~13 controls), BorderlineSMOTE generated approximately 11 synthetic control samples to achieve class balance. These synthetic samples were used solely for model training and were discarded at the end of each fold. The held-out test sample in every LOOCV iteration was an original, non-synthetic observation. Consequently, all performance metrics, including sensitivity, and specificity were computed exclusively from the 39 original participants, with no synthetic data contributing to any evaluation result.

### 2.4. Model Selection and Comparative Analysis

Five machine learning algorithms were evaluated to enable a systematic comparison across the interpretability–performance spectrum: Explainable Boosting Machine (EBM), Support Vector Machine (SVM), Logistic Regression (LR), Gradient Boosting Machine (GBM), and AdaBoost. These models were deliberately selected to span inherently interpretable architectures (EBM, LR) and black-box approaches (SVM, GBM, AdaBoost), allowing direct assessment of whether interpretability can be achieved without sacrificing predictive performance in a metabolomics classification task [9,17,18,19].

Explainable Boosting Machine (EBM): A glass-box generalized additive model (GAM) with automatic pairwise interaction detection that decomposes predictions into exact, visualisable shape functions: g(E[y]) = β_0_ + Σ_i_ f_i_(x_i_) + Σ_i__j_ f_i__j_(x_i_, x_j_), where g denotes the logistic link function, f_i_ the univariate shape function for metabolite i, and f_i__j_ the pairwise interaction term. These functions represent the model directly, not post hoc approximations of it. EBM was designated the primary model of interest on this basis; all remaining models served as comparators.

Support Vector Machine (SVM): Kernel-based classifier using a radial basis function (RBF) kernel. Probability estimates were obtained via Platt sigmoid calibration implemented through CalibratedClassifierCV (cv = 3, method = ‘sigmoid’), which was required to enable ROC-AUC and PR-AUC computation from pooled LOOCV probability scores.

Logistic Regression (LR): Included as the simplest reference model with global linear interpretability, providing a performance baseline against which nonlinear models can be evaluated.

Gradient Boosting Machine (GBM): The scikit-learn implementation of gradient boosting with decision tree base learners, using depth-wise tree growth and log-loss optimisation for binary classification.

Adaptive Boosting (AdaBoost): A meta-ensemble method that iteratively reweights training instances based on classification errors, using decision stumps or shallow trees as weak learners.

### 2.5. Hyperparameter Optimization

Hyperparameter optimisation was embedded within the outer LOOCV loop to prevent selection bias. In each of the 39 outer iterations, a randomised search with 3-fold stratified inner cross-validation was applied exclusively to the 38 training samples, with ROC-AUC as the optimisation criterion. Three-fold inner cross-validation was chosen over five-fold to preserve adequate class representation per fold given the limited training set size (~24 sepsis, ~13 controls per outer fold). The parameter search space explored for each model is presented in Table 1. For EBM, 13 candidate configurations were evaluated in the inner search, covering learning dynamics (learning_rate, max_rounds), feature resolution (max_bins), regularisation (min_samples_leaf), ensemble variance reduction (outer_bags), and interaction complexity (interactions). For the comparison models (SVM, Logistic Regression, Gradient Boosting, AdaBoost), the search space covered the principal regularisation and complexity parameters listed in Table 1. The hyperparameter configuration yielding the highest mean inner-fold ROC-AUC was selected and used to train the final model on all 38 training samples of that outer fold. This process was repeated independently for each of the 39 outer iterations; no parameter information was shared across outer folds. Hyperparameter stability cannot be definitively established at this sample size. The modal optimal values reported in Table 1 represent the most frequently selected configurations across the 39 outer folds; however, selection varied across folds, reflecting the inherent uncertainty of inner cross-validation with approximately 38 training samples per outer fold.

### 2.6. Model Evaluation

Model performance was assessed using leave-one-out cross-validation [20,21,22] (LOOCV; 39 iterations) within a fully nested framework. In each outer iteration, one participant was held out as the test sample, and the remaining 38 constituted the training set [23]. Preprocessing steps (log transformation, KNN imputation, StandardScaler) were applied to the full 38-sample outer training partition before inner cross-validation. The resulting pre-transformed training matrix was used as input to the 3-fold stratified inner cross-validation for hyperparameter selection. These preprocessing steps were therefore applied once per outer fold, not re-applied within each inner fold. BorderlineSMOTE was not applied within individual inner-fold splits. After hyperparameter selection, BorderlineSMOTE was applied to the full 38-sample pre-transformed outer training partition to achieve class balance before final model training. The held-out test sample was transformed using the training-fold-fitted imputer and scaler, and evaluated against the trained model without any exposure to fitting, SMOTE, or hyperparameter selection steps. Consequently, all data-dependent decisions—including preprocessing, class balancing, and hyperparameter tuning—are confined to the training partition of each outer fold. The following steps were executed sequentially and entirely within the training partition of each outer fold:

- Log_1_(x + 1) transformation of all 22 features.
- KNN imputation (k = 5), fit on training samples only.
- StandardScaler normalisation, fit on training samples only.
- Hyperparameter optimisation via 3-fold stratified inner cross-validation on the training samples.
- BorderlineSMOTE (k_neighbors = 3) applied to the training samples after inner cross-validation, using the selected optimal hyperparameters, to balance class distribution prior to final model fitting.

Final model training on the SMOTE-augmented training fold using the selected hyperparameters. The held-out test sample was transformed using the training-fold-fitted imputer, scaler, and evaluated against the trained model without any exposure to fitting, SMOTE, or hyperparameter selection steps. This design ensures that all data-dependent decisions, including class balancing, and hyperparameter tuning are strictly confined to the training partition of each outer fold, with no information from the test sample influencing any stage of model development.

### 2.7. Sensitivity Analysis

To assess whether BorderlineSMOTE contributed to performance inflation, a parallel sensitivity analysis was conducted using an identical nested LOOCV framework in which synthetic oversampling was omitted entirely. Class imbalance was addressed using cost-sensitive learning: inverse-frequency sample weights were computed at each outer fold using compute_sample_weight(class_weight = ‘balanced’) applied exclusively to the 38 training samples of that fold, and passed to each model’s fit() method. This approach up-weights minority class instances during loss optimisation without modifying the training dataset and is supported by all five evaluated models. No synthetic observations were generated at any stage. As in the primary analysis, all test predictions derive from original, non-synthetic participants, and all performance metrics are computed exclusively from the 39 original participants. Results are presented in Supplementary Table S1.

### 2.8. Performance Evaluation

Model performance was comprehensively assessed using the following metrics: accuracy, F1-score, sensitivity, specificity, area under the receiver operating characteristic curve (ROC-AUC), Precision-Recall AUC (PR-AUC), and Brier probability score. PR-AUC was included alongside ROC-AUC because it provides a more informative measure of performance under class imbalance (25 positives vs. 14 negatives), as it does not account for true negatives and directly reflects model precision across recall thresholds. 95% confidence intervals were estimated using 2000-iteration stratified bootstrap resampling.

### 2.9. Explainable Boosting Machine Framework

Among the evaluated algorithms, EBM offers a distinctive advantage by combining state-of-the-art predictive performance with inherent interpretability. Built upon the Generalized Additive Model (GAM) framework, EBM decomposes predictions into individual variable contributions: g(E[y]) = β_0_ + Σ_i_ f_i_(x_i_) + Σ_i__j_ f_i__j_(x_i_, x_j_), where g represents the logistic link function, f_i_ denotes univariate shape functions capturing non-linear metabolite effects, and f_i__j_ represents pairwise interaction terms [24,25]. We extracted both global feature importance rankings (aggregated across all samples) and local explanations (individual patient-level predictions) to identify the most discriminative platelet metabolites and elucidate their contributions to sepsis pathophysiology.

### 2.10. Statistical Analysis

Sex distribution was compared using Fisher’s exact test and is reported as frequency (percentage). Age was compared using the independent samples *t*-test and is reported as mean ± standard deviation (SD). Statistical significance was set at *p* < 0.05.

### 2.11. Statistical Computing Environment

All machine learning analyses were performed in Python 3.12 using scikit-learn 1.5.2 (model training, cross-validation, calibration), interpret 0.7.8 (EBM implementation and interpretability), imbalanced-learn 0.12.4 (BorderlineSMOTE), pandas 2.2.3, numpy 1.26.4, and matplotlib 3.9.2. Code is available on GitHub (https://github.com/drhilal/Sepsis-study; accessed on 15 May 2026).

## 3. Results

The patients participating in the study showed a statistically similar distribution according to their gender (Fisher’s exact test, *p* = 0.862). Sex distribution did not differ significantly between groups: 8 males (57.1%) and 6 females (42.9%) in the non-sepsis group versus 15 males (60.0%) and 10 females (40.0%) in the sepsis group. Mean age was 44 ± 14 years in controls and 55 ± 17 years in sepsis patients (*p* = 0.056; standardised mean difference (SMD) = 0.69). Although the age difference did not reach conventional statistical significance, the 11-year difference represents a moderate effect size (SMD = 0.69) that may independently influence platelet metabolite concentrations.

Performance metrics for all five models evaluated using LOOCV on the 39 original participants are summarised in Table 2. Reported performance reflects predictions on the 39 original test samples; BorderlineSMOTE was applied exclusively within training folds and does not affect evaluation data. EBM achieved the highest ROC-AUC (0.864; 95% CI: 0.736–1.000) and PR-AUC (0.902; 95% CI: 0.783–0.997) among all evaluated models, with sensitivity 0.880 (TP = 22/25), specificity 0.714 (TN = 10/14), accuracy 0.821, and F1-score 0.863. AdaBoost ranked second by ROC-AUC (0.779; 95% CI: 0.595–0.980) and by PR-AUC (0.859; 95% CI: 0.739–0.957). The confidence intervals of all models overlap substantially, indicating that no pairwise performance difference is statistically meaningful at n = 39. EBM is preferred on the basis of its highest point estimates across all metrics and its inherent glass-box interpretability, which no other evaluated model provides without post hoc approximation. SVM (ROC-AUC = 0.676) and LR (ROC-AUC = 0.649) performed below the ensemble methods on all metrics.

Supplementary Table S1 presents model performance under the SMOTE-free pipeline. Comparing primary and sensitivity analyses, BorderlineSMOTE had a modest and model-dependent effect. For EBM, removal of BorderlineSMOTE reduced sensitivity from 0.880 to 0.800, while specificity remained unchanged, resulting in a reduction in ROC-AUC from 0.864 to 0.821 and PR-AUC from 0.902 to 0.881. For GBM, a marginal reduction was observed. For SVM and Logistic Regression, confusion matrices were identical across both conditions, indicating that cost-sensitive weighting alone was sufficient to achieve equivalent classification thresholds for these models. AdaBoost showed negligible differences. These findings indicate that BorderlineSMOTE did not produce substantial performance inflation: the reduction in EBM sensitivity upon SMOTE removal (two fewer correct sepsis classifications) is consistent with the expected role of borderline synthetic samples in improving discrimination near the decision boundary rather than artificially inflating overall performance. EBM retained the highest ROC-AUC and PR-AUC among all models in both the primary and sensitivity analyses.

**Figure 1:**
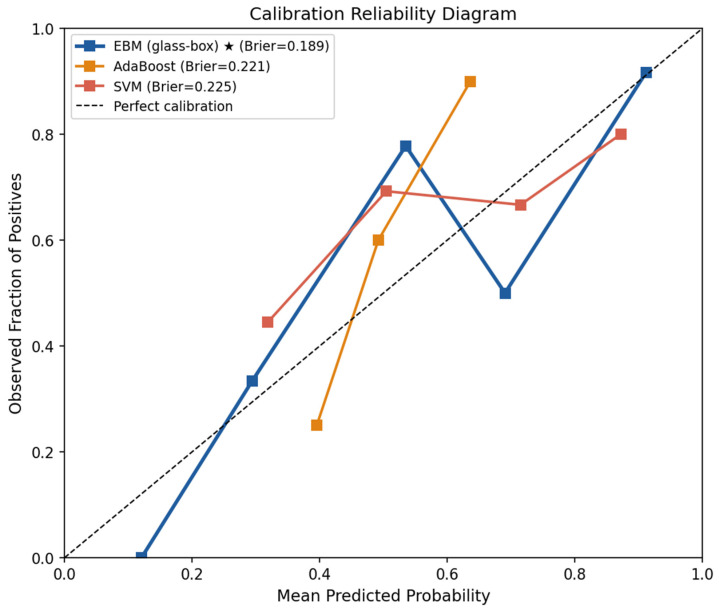
Reliability graph for the three best calibrated models; *: optimal model.

In addition, EBM demonstrated a Brier score of 0.189, outperforming AdaBoost (0.221), SVM (0.225), Logistic Regression (0.272), and Gradient Boosting (0.290). Lower Brier scores indicate better calibration of predicted probabilities. Reliability diagrams for the three best-calibrated models are presented in Figure 1.

**Figure 2:**
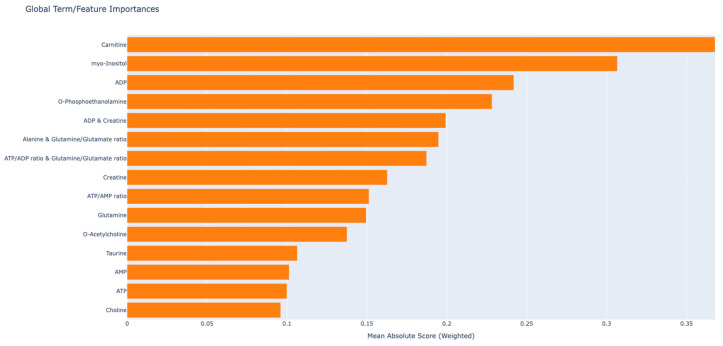
Global term and feature importance rankings from the EBM model. The x-axis represents the mean absolute contribution score (weighted); higher values indicate greater overall discriminative contribution. Orange bars represent single-feature terms; terms containing ‘&’ denote automatically detected pairwise interaction terms. ADP = adenosine diphosphate; ATP = adenosine triphosphate; AMP: adenosine monophosphate.

Global feature importance analysis of the EBM model identified Carnitine as the single most influential metabolite, with a mean absolute contribution score of 0.368, followed by myo-Inositol (0.307), ADP (0.242), and O-Phosphoethanolamine (0.228) (Figure 2). These four features accounted for the dominant portion of the model’s discriminative signal and each represent established pathways in sepsis pathophysiology. Beyond individual metabolites, three pairwise interaction terms appeared among the ten highest-ranked features: ADP & Creatine (0.199), Alanine & Glutamine/Glutamate ratio (0.195), and ATP/ADP ratio & Glutamine/Glutamate ratio (0.187). The detection of these interactions by EBM underscores the non-additive relationships between energy metabolism and amino acid homeostasis in the septic state—relationships that univariate feature importance methods or linear models cannot capture. O-Acetylcholine ranked eleventh (0.138), consistent with cholinergic anti-inflammatory pathway dysfunction during sepsis, wherein impaired vagal signalling fails to suppress macrophage activation and pro-inflammatory cytokine production. Taurine (0.106), an osmoregulatory and antioxidant metabolite, and the ATP/AMP ratio (0.151), a surrogate for AMPK activation, provided additional discriminative signal reflecting the oxidative and metabolic stress characteristic of critical illness.

**Figure 3:**
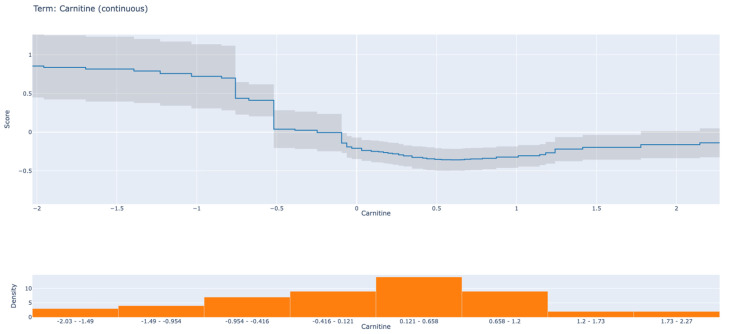
EBM shape function for Carnitine. (**Top**): the blue line represents the learned non-linear relationship between standardised log-transformed Carnitine concentration (*x*-axis) and the EBM contribution score (*y*-axis; logit scale). The grey shaded band denotes the 95% uncertainty interval derived from outer-bag variance. (**Bottom**): frequency distribution (density) of observed Carnitine values across the 39 participants, with bin ranges labelled on the *x*-axis.

The Carnitine shape function revealed a predominantly inverse relationship between platelet carnitine concentration and the sepsis prediction score (Figure 3). At concentrations more than one standard deviation below the cohort mean, a range that included a notable proportion of sepsis patients, as shown in the density histogram, the EBM contribution score approached +1.0, indicating strong positive support for sepsis classification. As carnitine concentration increased through the intermediate range (approximately −0.5 to 0 on the standardised scale), the score declined sharply, crossing zero and becoming negative at concentrations above the mean, where contributions of approximately −0.3 to −0.5 were observed. A slight upward inflection appeared at the highest concentrations (>1.5 SD), though the wide confidence intervals in this sparsely populated region preclude reliable interpretation. The confidence bands were narrowest across the central high-density region (−0.416 to 0.658), where model estimates were most reliable.

**Figure 4:**
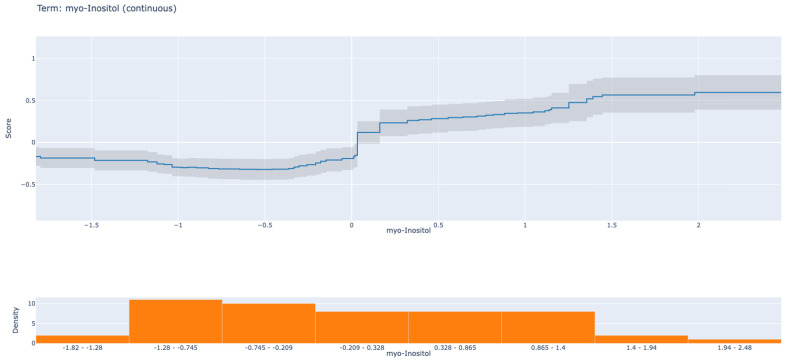
EBM shape function for myo-Inositol. (**Top**): the blue line represents the learned non-linear relationship between standardised log-transformed myo-Inositol concentration (*x*-axis) and the EBM contribution score (*y*-axis; logit scale). The grey shaded band denotes the 95% uncertainty interval from outer-bag variance. (**Bottom**): frequency distribution of observed myo-Inositol values across the 39 participants.

The myo-Inositol shape function demonstrated a broadly positive relationship between concentration and sepsis prediction score, with a clear inflection at approximately zero on the standardised scale (Figure 4). Below this threshold, contribution scores were mildly negative (approximately −0.2 to −0.3), indicating a modest association with the non-sepsis class at low concentrations. Above zero, the score rose monotonically, reaching values between +0.3 and +0.6 at higher concentrations, with the trend continuing through the uppermost range. The density histogram confirmed that observations were distributed across both sides of this inflection, with the largest concentration of samples in the −1.28 to 0.328 region. Confidence intervals were appropriately narrow in this high-density zone and widened at both extremes.

**Figure 5:**
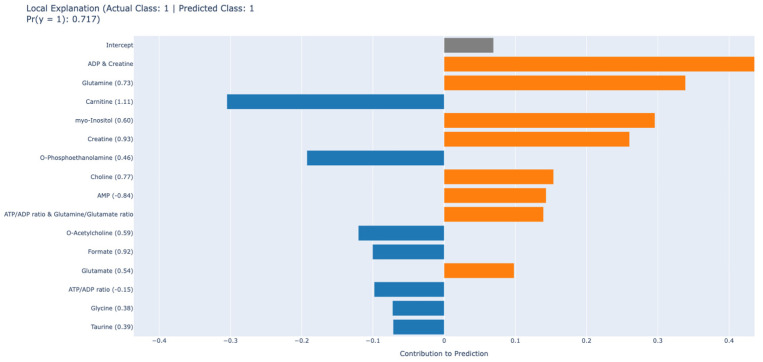
Local EBM explanation for a representative true positive case (Actual Class: 1 [Sepsis]|Predicted Class: 1 [Sepsis]|Pr(y = 1) = 0.717). Orange bars indicate positive contributions toward sepsis classification; blue bars indicate negative contributions toward non-sepsis classification. Values in parentheses denote the standardised metabolite concentration for this participant. The intercept (grey bar) represents the population-level baseline.

Figure 5 presents the local additive EBM explanation for a correctly classified sepsis patient. The predicted probability of 0.717 resulted from the balance of opposing metabolite contributions, with positive signals ultimately outweighing negative signals. The dominant positive contribution was the ADP and Creatine binary interaction term (+0.43), suggesting that the co-occurrence of specific ADP and Creatine concentration levels in this patient generated a synergistic signal that outweighed the contribution of each metabolite alone. This interaction likely captures a coordinated signature of purine nucleotide depletion and impaired phosphocreatine-mediated energy buffering; both features of sepsis-induced mitochondrial dysfunction. Glutamine (+0.35) and myo-inositol (+0.32) provided the next largest positive contributions, consistent with population-level shape functions for these metabolites at above-average concentrations. Creatine (+0.30) and Choline (+0.14) contributed moderately in the same direction. Specifically, this patient’s carnitine level showed the largest single negative contribution (−0.30), reducing the model’s sepsis class probability. The overall prediction value of 0.717 demonstrates that EBM correctly identified this patient as having sepsis despite atypical carnitine levels by integrating the convergent evidence from ADP-Creatine interaction, Glutamine, and myo-Inositol into a prediction exceeding the classification threshold.

## 4. Discussion

The present study demonstrates the feasibility of an explainable machine learning approach integrating platelet metabolomics for sepsis prediction in a proof-of-concept framework. EBM model achieved the highest ROC-AUC (0.864; 95% CI: 0.736–1.000), PR-AUC (0.902), and Brier score (0.189) among all evaluated models, with sensitivity 0.880 (TP = 22/25) and specificity 0.714 (TN = 10/14), while providing mechanistically interpretable feature contributions at both global and individual patient levels. These findings advance the field of sepsis biomarker research by establishing a methodological framework that bridges sophisticated predictive modeling with clinically meaningful metabolic insights. However, these results should be interpreted as exploratory: a model trained and internally validated using LOOCV on 39 individuals from a single centre, without external validation, cannot be positioned as ready for clinical deployment. The present results establish a methodological foundation and generate mechanistic hypotheses that warrant prospective investigation in larger, independent, multi-centre cohorts before any clinical application is considered.

The superior discriminative performance of EBM relative to SVM, Logistic Regression, GBM and AdaBoost merits consideration from both technical and clinical perspectives. While GBM and AdaBoost demonstrated comparable ROC-AUC values (0.765 and 0.779, respectively), EBM’s glass-box architecture enables clinicians to examine precisely how metabolite values translate into prediction scores—a transparency that black-box ensemble methods cannot intrinsically provide. Previous studies applying machine learning to sepsis prediction have achieved similar performance levels using gradient boosting approaches [7,8,26], yet the interpretability dimension has typically been addressed through post hoc techniques such as SHAP rather than through inherently interpretable model architectures. Our results suggest that inherent interpretability need not come at the cost of predictive accuracy, supporting the broader adoption of glass-box models in clinical applications where understanding the “why” behind predictions is as important as the predictions themselves [27,28]. The distinction between inherent and post hoc interpretability carries important implications for clinical deployment. While SHAP and LIME provide valuable approximations of feature contributions, these explanations represent local linear approximations that may not faithfully represent the underlying model behavior, particularly in regions of high non-linearity or feature interaction. EBM’s glass-box architecture ensures that the visualized shape functions exactly represent the learned relationships, eliminating the potential for misleading explanations that could compromise clinical decision-making [29].

The current findings should be interpreted in the context of a previous study that implemented a similar XAI-based metabolomic framework [13]. The most significant methodological difference relates to the validation design. The previous study applied SMOTE-Tomek oversampling, hyperparameter optimization, and model selection on the entire dataset prior to 5-fold cross-validation assessment. This design allows synthetic observations and hyperparameter selections to draw information from the full sample, including observations used for testing later, a known source of selection optimism. In this study, a fully nested framework was implemented: log transform, KNN assignment, StandardScaler normalization, hyperparameter optimization via 3-fold internal cross-validation, and BorderlineSMOTE class adjustment were applied specifically within the training section of each outer LOOCV layer. The separated test sample did not affect any of the preprocessing, class adjustment, or model selection steps. Performance estimates in this study are significantly more conservative, consistent with the known impact of nested and non-nested validation in small sample settings. We believe these estimates are less biased representations of true out-of-sample performance. A second point concerns the interpretability framework. The previous study used SHAP values applied later to XGBoost and LightGBM outputs. While SHAP provides valuable approximations of feature contributions, these values represent local linear approximations that may deviate from true model behavior in regions of high nonlinearity or feature interaction; this is an inherent limitation of the later explanation of black-box models. In this study, EBM was identified as the primary model because its shape functions and local additive decompositions are complete representations of the learned model, not post hoc approximations. The distinction between natural and later interpretability has direct implications for clinical confidence and regulatory transparency. Additional methodological advancements in this study include: the introduction of three biologically motivated metabolite ratio features (ATP/ADP, ATP/AMP, Glutamine/Glutamate) not evaluated in the previous study; and the examination of measures addressing class imbalance with PR-AUC as well as ROC-AUC, and an evaluation of the Brier score and calibration curves, which indicate how well calibrated the predictions of all models are.

The prominence of carnitine and glutamine among discriminative metabolites resonates with established knowledge of sepsis-induced metabolic reprogramming and offers potential therapeutic implications. Carnitine, essential for shuttling long-chain fatty acids into mitochondria for β-oxidation, exhibits significant alterations in sepsis that correlate with disease severity and outcomes. Puskarich et al. [6] identified acetylcarnitine as a superior predictor of 90-day sepsis mortality compared to lactate—outperforming this traditional marker even after adjustment for age and SOFA score—and demonstrated that carnitine supplementation may benefit specific subgroups of septic shock patients. Our findings extend these observations by demonstrating that carnitine derivatives contribute substantially to sepsis classification at the diagnostic stage, suggesting continuity between early metabolic perturbations and later prognostic trajectories that could inform both diagnosis and targeted therapeutic interventions.

myo-Inositol, the second most discriminative metabolite (mean absolute contribution = 0.307), demonstrated a broadly positive relationship with sepsis classification in its shape function concentrations above the cohort mean were associated with increasing EBM contribution scores (+0.3 to +0.6), while below-average concentrations contributed modestly toward the non-sepsis class. myo-Inositol participates in phosphoinositide signalling pathways that regulate immune cell activation, platelet aggregation, and endothelial permeability. Its elevation in septic platelets is consistent with activation of inflammatory signalling cascades and altered phospholipid remodelling during systemic infection. The detection of myo-Inositol as a leading discriminative feature supports the hypothesis that platelet phosphoinositide metabolism is substantially reprogrammed during sepsis, potentially contributing to the coagulation-inflammation interface characteristic of the septic state.

ADP (third-ranked, score = 0.242) and O-Phosphoethanolamine (fourth-ranked, score = 0.228) further underscore the centrality of purine nucleotide and phospholipid metabolism in sepsis pathophysiology. ADP is both a product of ATP hydrolysis during bioenergetic stress and a potent platelet activator through P2Y1 and P2Y12 receptors; its elevation in septic platelets reflects concurrent ATP depletion and platelet hyper-reactivity. O-Phosphoethanolamine is an intermediate in the Kennedy pathway for phosphatidylethanolamine biosynthesis; its altered concentration in sepsis is consistent with the widespread remodelling of membrane phospholipid composition that accompanies systemic inflammatory activation and platelet dysfunction. These features also contributed prominently to the three highest-ranked pairwise interaction terms: ADP–Creatine (0.199), Alanine–Glutamine/Glutamate ratio (0.195), and ATP/ADP–Glutamine/Glutamate ratio (0.187), demonstrating that non-additive energy–amino acid metabolic relationships carry additional discriminative information beyond the individual features alone. The Alanine–Glutamine/Glutamate ratio interaction (0.195) is particularly noteworthy: alanine participates in the glucose–alanine cycle and serves as a major gluconeogenic substrate; its co-variation with the Glutamine/Glutamate ratio likely reflects the interplay between amino acid catabolism, gluconeogenic demand, and redox buffering during sepsis-induced metabolic reprogramming [30].

O-Acetylcholine ranked eleventh in global feature importance (mean absolute contribution = 0.138), consistent with cholinergic anti-inflammatory pathway (CAP) dysfunction during sepsis. The vagus nerve-mediated inflammatory reflex, operating through α7 nicotinic acetylcholine receptors on immune cells, suppresses macrophage activation and pro-inflammatory cytokine release under physiological conditions. The inverse relationship between platelet O-Acetylcholine concentration and sepsis prediction score observed in the shape function, wherein low levels contribute positively to sepsis classification, is consistent with CAP dysfunction during systemic inflammation. Platelet-derived acetylcholine metabolites may therefore serve as accessible biomarkers reflecting systemic cholinergic tone and vagal regulatory capacity, with potential implications for emerging bioelectronic or pharmacological CAP-targeting therapies [31,32,33].

Taurine (mean absolute contribution = 0.106) and the ATP/AMP ratio (0.151) contributed additional discriminative signal reflecting the oxidative stress and bioenergetic dysfunction characteristic of critical illness. Taurine, an endogenous osmoregulatory and antioxidant molecule synthesised from cysteine, is abundant in platelets and plays a cytoprotective role under conditions of oxidative challenge [34]. Its depletion in sepsis likely reflects excessive consumption in response to mitochondrial reactive oxygen species and lipid peroxidation, consistent with the pan-oxidative phenotype of the septic state [35,36]. The ATP/AMP ratio, a direct indicator of cellular energy charge and AMPK activation status, captures the bioenergetic imbalance imposed by sepsis-induced cytopathic hypoxia: impaired oxidative phosphorylation reduces ATP synthesis while AMP accumulates, activating AMPK as a compensatory metabolic switch [37,38]. The fact that this ratio appears among the top features independently of the raw ADP value underscores the additive information carried by derived metabolite ratios beyond individual metabolite concentrations, validating the biologically motivated feature engineering strategy employed in this study.

The local interpretation analyses provide clinically meaningful insights beyond aggregate feature importance. The true positive case (Figure 5; Pr(y = 1) = 0.717) exemplifies the canonical sepsis metabolic phenotype: the dominant positive contribution was the ADP–Creatine binary interaction term (+0.43), reflecting a coordinated signature of purine nucleotide depletion and impaired phosphocreatine-mediated energy buffering—both hallmarks of sepsis-induced mitochondrial dysfunction [37,38,39,40]. Glutamine (+0.35) and myo-Inositol (+0.32) provided the next largest positive contributions, followed by Creatine (+0.30) and Choline (+0.14). This patient’s carnitine level constituted the largest single negative contribution (−0.30), illustrating how EBM integrates convergent metabolic evidence to classify correctly despite atypical values in individual features. These locally interpretable patterns are directly traceable to the global shape functions and interaction scores, ensuring biological consistency between population-level and patient-level explanations.

Several limitations warrant acknowledgment and should inform interpretation of our findings and design of future studies. First, the modest sample size (n = 39) constrains statistical power and generalizability, as reflected in the relatively wide confidence intervals surrounding performance estimates. While leave-one-out cross-validation maximizes data utilization, external validation in larger, independent, multi-center cohorts is essential before clinical implementation. Second, the cross-sectional design captures metabolic status at a single time point, precluding assessment of temporal dynamics that may enhance both diagnostic and prognostic utility. Longitudinal sampling could reveal metabolic trajectories that discriminate sepsis from non-septic inflammation more reliably than static measurements and potentially enable earlier detection. Third, non-sepsis control group (ED patients without platelet-affecting conditions) may not fully represent the clinical population in which sepsis prediction is most relevant and challenging. Future studies should compare sepsis patients against those with SIRS from non-infectious causes, non-septic critical illness, and localized infections without systemic involvement to establish diagnostic specificity in clinically challenging scenarios where differentiation is most needed. Fourth, while NMR spectroscopy offers excellent reproducibility and absolute quantification, its sensitivity for low-abundance metabolites is inferior to mass spectrometry platforms, potentially limiting discovery of additional discriminative markers with lower physiological concentrations [34]. Integration of platelet metabolomics with established clinical biomarkers such as SOFA score components, ferritin, and novel proteomics-derived signatures [30,41] in future multi-modal prediction frameworks may substantially enhance discriminative performance and clinical utility. Furthermore, with approximately 38 training samples per outer fold, inner cross-validation hyperparameter estimates carry substantial uncertainty; while performance across near-optimal EBM configurations was relatively stable, the selected configuration may not represent a globally stable optimum, and this instability is an acknowledged limitation of hyperparameter optimisation in small-sample settings. Additionally, age was not directly adjusted for in the predictive modelling framework. The 11-year age difference between groups (SMD = 0.69, *p* = 0.056) represents a potential confound, as platelet metabolite concentrations may vary independently with age through age-related mitochondrial dysfunction, altered energy metabolism, and changes in platelet reactivity. This introduces uncertainty in whether the identified metabolic signatures reflect sepsis-specific pathophysiology, age-related differences, or an interaction between the two. Future studies should employ age-balanced recruitment strategies and/or include age as a covariate in sensitivity analyses to disentangle sepsis-specific metabolic signatures from age-related metabolic variation.

Despite these limitations, the present study establishes a foundation for interpretable metabolomics-based sepsis prediction that addresses critical barriers to clinical translation. Future directions should encompass: (1) larger, prospective, multi-center validation studies with appropriate comparator groups; (2) integration of metabolomic signatures with established clinical scoring systems such as SOFA and qSOFA to create hybrid prediction models; (3) development of point-of-care NMR or mass spectrometry platforms enabling rapid metabolite quantification at the bedside; (4) exploration of metabolomic-guided therapeutic interventions targeting specific metabolic derangements identified as drivers of individual patient predictions; and (5) investigation of whether metabolic phenotyping can identify sepsis endotypes with differential treatment responses. Additionally, the integration of metabolomic signatures with multi-omics data—including transcriptomics, proteomics, and genomics—may enhance predictive accuracy and provide deeper mechanistic insights into sepsis endotypes [42]. The development of federated learning approaches could enable model training across multiple institutions while preserving data privacy, facilitating the creation of more generalizable prediction models. Finally, prospective clinical trials evaluating whether metabolomics-guided clinical decision support improves patient outcomes compared to standard care are needed to establish clinical utility and cost-effectiveness.

## 5. Conclusions

This study demonstrates that platelet metabolomics integrated with explainable machine learning provides a promising proof-of-concept framework for interpretable sepsis prediction. The Explainable Boosting Machine achieved the highest discriminative performance (ROC-AUC = 0.864; 95% CI: 0.736–1.000) while enabling transparent examination of feature contributions at both global and individual patient levels. Carnitine, myo-Inositol, ADP, and O-Phosphoethanolamine emerged as the most discriminative platelet metabolites, reflecting metabolic reprogramming and energy metabolism disruption characteristic of sepsis pathophysiology. Pairwise interaction terms (ADP–Creatine, Alanine–Glutamine/Glutamate ratio, ATP/ADP–Glutamine/Glutamate ratio) further highlighted non-additive relationships between energy and amino acid metabolism that univariate methods cannot capture. Local interpretations provided patient-level mechanistic insights that complement aggregate diagnostic accuracy. These findings position the EBM framework as a promising hypothesis-generating tool that requires external validation in larger, independent, multi-center cohorts before any clinical application is considered.

## Figures and Tables

**Table 1:** Hyperparameter Search Space and Optimal Values Identified.

Model	Parameter	Search Space	Optimal Value
EBM	learning_rate	[0.001, 0.003, 0.005, 0.008, 0.010, 0.020, 0.050]	0.010
	max_bins	[64, 128, 256]	256
	min_samples_leaf	[1, 2, 3, 5]	1
	outer_bags	[8, 12, 16, 20]	16
	max_rounds	[100, 150, 200, 250, 300, 400, 500]	200
	interactions	[0, 3, 5, 7]	5
SVM	C	[0.01, 0.1, 1.0, 10, 100]	1.0
	kernel	[rbf, linear, poly]	rbf
	gamma	[scale, auto, 0.001, 0.01, 0.1]	scale
LR	C	[0.001, 0.01, 0.1, 1.0, 10, 100]	1.0
	penalty	[l1, l2]	l2
GBM	learning_rate	[0.01, 0.05, 0.1, 0.2]	0.05
	max_depth	[3, 5, 7, 10]	3
	n_estimators	[50, 100, 200, 500]	100
	subsample	[0.6, 0.8, 1.0]	1.0
AdaBoost	learning_rate	[0.01, 0.1, 0.5, 1.0, 1.5]	0.5
	n_estimators	[50, 100, 200, 500]	100

Note: Modal optimal value represents the most frequently selected configuration across the nested LOOCV outer folds.

**Table 2:** Performance metrics for all five models (LOOCV, n = 39, default threshold = 0.50). 95% CIs from 2000-iteration stratified bootstrap resampling.

Model	TP	TN	FP	FN	Sensitivity	Specificity	Accuracy	F1-Score	ROC-AUC	PR-AUC	Brier
EBM	22	10	4	3	0.880 (0.640–1.000)	0.714 (0.357–1.000)	0.821 (0.692–0.923)	0.863 (0.744–0.949)	0.864 (0.736–1.000)	0.902 (0.783–0.997)	0.189 (0.130–0.258)
SVM	17	8	6	8	0.680 (0.440–0.920)	0.571 (0.214–0.929)	0.641 (0.487–0.795)	0.708 (0.545–0.840)	0.676 (0.421–0.872)	0.812 (0.674–0.928)	0.225 (0.171–0.286)
LR	16	8	6	9	0.640 (0.400–0.880)	0.571 (0.214–0.929)	0.615 (0.462–0.769)	0.681 (0.500–0.821)	0.649 (0.383–0.857)	0.799 (0.650–0.919)	0.272 (0.206–0.343)
GBM	19	9	5	6	0.760 (0.520–1.000)	0.643 (0.286–1.000)	0.718 (0.564–0.846)	0.776 (0.625–0.885)	0.765 (0.577–0.933)	0.853 (0.723–0.955)	0.290 (0.175–0.414)
AdaBoost	18	10	4	7	0.720 (0.480–0.960)	0.714 (0.357–1.000)	0.718 (0.564–0.846)	0.766 (0.615–0.885)	0.779 (0.595–0.980)	0.859 (0.739–0.957)	0.221 (0.198–0.245)

TP/TN/FP/FN = true/false positives/negatives; ROC-AUC = area under ROC curve; PR-AUC = area under Precision-Recall curve.

## Data Availability

The raw data supporting the conclusions of this article will be made available by the authors on request.

## Supplementary Materials

The following supporting information can be downloaded at: https://www.mdpi.com/article/10.3390/diagnostics16111643/s1, Table S1: Sensitivity analysis performance without BorderlineSMOTE.
