# Supplementary material for: Explainable Boosting Machine in Sepsis Prediction Using Platelet Metabolomics: An Interpretable Machine Learning Approach
Source: Diagnostics (Basel). 2026 May 27;16(11):1643. doi: 10.3390/diagnostics16111643 (PMC13256616; doi:10.3390/diagnostics16111643)
Supplement: Supplementary file 1 [file diagnostics-16-01643-s001.zip › diagnostics-4187697-supplementary.pdf]

Table S1. Sensitivity analysis performance without BorderlineSMOTE

Model	TP	TN	FP	FN	Sensitivity	Specificity	Accuracy	F1	ROC-AUC	PR-AUC
EBM	20	10	4	5	0.800	0.714	0.769	0.816	0.821	0.881
SVM	17	8	6	8	0.680	0.571	0.641	0.708	0.676	0.812
LR	16	8	6	9	0.640	0.571	0.615	0.681	0.649	0.799
GBM	18	9	5	7	0.720	0.643	0.692	0.750	0.741	0.841
AdaBoost	19	10	4	6	0.760	0.714	0.744	0.792	0.800	0.870
